# Fabrication of Hierarchical Layer-by-Layer Assembled Diamond-based Core-Shell Nanocomposites as Highly Efficient Dye Absorbents for Wastewater Treatment

**DOI:** 10.1038/srep44076

**Published:** 2017-03-08

**Authors:** Xinna Zhao, Kai Ma, Tifeng Jiao, Ruirui Xing, Xilong Ma, Jie Hu, Hao Huang, Lexin Zhang, Xuehai Yan

**Affiliations:** 1 State Key Laboratory of Metastable Materials Science and Technology, Yanshan University, Qinhuangdao 066004, China; 2 Hebei Key Laboratory of Applied Chemistry, School of Environmental and Chemical Engineering, Yanshan University, Qinhuangdao 066004, China; 3 State Key Laboratory of Biochemical Engineering, Institute of Process Engineering, Chinese Academy of Sciences, Beijing 100190, China

## Abstract

The effective chemical modification and self-assembly of diamond-based hierarchical composite materials are of key importance for a broad range of diamond applications. Herein, we report the preparation of novel core-shell diamond-based nanocomposites for dye adsorption toward wastewater treatment through a layer-by-layer (LbL) assembled strategy. The synthesis of the reported composites began with the carboxyl functionalization of microdiamond by the chemical modification of diamond@graphene oxide composite through the oxidation of diamond@graphite. The carboxyl-terminated microdiamond was then alternatively immersed in the aqueous solution of amine-containing polyethylenimine and carboxyl-containing poly acrylic acid, which led to the formation of adsorption layer on diamond surface. Alternating (self-limiting) immersions in the solutions of the amine-containing and carboxyl-containing polymers were continued until the desired number of shell layers were formed around the microdiamond. The obtained core-shell nanocomposites were successfully synthesized and characterized by morphological and spectral techniques, demonstrating higher surface areas and mesoporous structures for good dye adsorption capacities than nonporous solid diamond particles. The LbL-assembled core-shell nanocomposites thus obtained demonstrated great adsorption capacity by using two model dyes as pollutants for wastewater treatment. Therefore, the present work on LbL-assembled diamond-based composites provides new alternatives for developing diamond hybrids as well as nanomaterials towards wastewater treatment applications.

Nowadays, diamond is well known among scientists and technologists for its excellent mechanical and optical properties, high surface areas and thermal conductivity, special chemical and electrochemical stability, and tunable surface structures^1^,^2^,^3^,^4^. Research on diamond in terms of preparation, properties, and applications has become one of the hot spots in the field of nanomaterials and carbon materials. In recent years, much effort has been made to the rational design and controlled preparation of various diamond-based organic/inorganic composites, which display better electronic, optical, and electrochemical properties for nanomaterial applications. The chemical modification and functionalization of diamond composite materials with different compounds or nanoparticles are frequently needed to make them appropriate for good dispersion or with suitable function in various applications. For examples, various organic molecules including redox active ferrocene moiety^5^, long-chain fluorinated polymer^6^, poly(epsilon-caprolactone) (PCL)^7^, triethylene glycol (EG) and azide (−N_3_) groups^8^, can be used to modify diamond to obtain surface grafted diamond by various chemical reactions. Interestingly, the modification of diamond powder with fluorinated or aminated surfaces as well as special core-shell structures (diamond as core and TiN as shell) were beneficial to the next applications as electrocatalysts^9^,^10^. In addition, the diamond surface can be also modified with graphite or graphene to from all-carbon-based composite materials for subsequent functionalization and applications. For examples, Wang *et al*. synthesized the surface graphitized nanodiamond (GND) with a diamond core covered by a graphitic carbon shell through annealing ND at the temperature of 1300 °C in a vacuum of 10^−3^ Pa as a support for PtNi electrocatalysts^11^. Zhao *et al*. reported the preparation of Pt/TiO_2_/GND electrocatalysts from graphitized GND and TiO_2_ modified on Pt nanoparticles using a microwave hydrolysis method^12^. Wu *et al*. reported the Co-N-C/ND composite with a nanodiamond (ND) core covered with bifunctional cobalt-embedded nitrogen doped graphitized carbon shell as electrocatalysts for the oxygen reduction reaction and oxygen evolution reaction in alkaline media^13^. In addition, diamond/graphene composites have been also prepared by electron-beam (E-beam) irradiation or annealing in a vacuum at high temperatures^14^,^15^,^16^. Hence, it represents a formidable challenge to effectively prepare diamond-based composites with organized self-assembled nanostructures.

The layer-by-layer (LbL) self-assembly technique is regarded as a simple and effective surface modification technology. The common driving forces of the layer assembly process are electrostatic interaction, hydrogen bond, coordination bond, π-π stacking, hydrophobic interaction and molecular recognition^17^,^18^,^19^,^20^,^21^,^22^,^23^,^24^. The LbL assembly technique has many advantages, such as simple process and mild preparation condition. It can be expected that a combination of diamond-based composites involved in diamond modification and LbL self-assembled technique should be particularly advantageous owing to their excellent biocompatibility, moderate nanostructures, and enhanced mechanical and chemical properties. Up to now, various water soluble molecules including poly(allylamine hydrochloride), zinc porphyrin complex, dipeptide (Phe-Lys) conjugated doxorubicin hydrochloride (DOX), protein bovine serum albumin (BSA), conductive polymers (polyaniline, polypyrrole, polythiophene and polyazopyridine), poly(diallyldimethylammonium chloride), have been reported to modify diamond surface via the LbL method for controlled drug release and other analytical applications^25^,^26^,^27^,^28^,^29^,^30^,^31^,^32^,^33^. However, it is still challenging to design and obtain diamond-based composites with facile and effective process in LbL self-assembled technique.

In the work presented here, we reported the synthesis and characterization of functionalized core-shell diamond composites made by LbL assembled strategy via amine-containing polyethylenimine (PEI) and carboxyl-containing poly acrylic acid (PAA). The carboxyl-terminated microdiamonds as building blocks originated from oxidation and chemical modification of initial commercial microdiamond. We investigated the characteristic core-shell structures by using various morphological and spectral characterization methods. The as-prepared mesoporous core-shell composites with the desired number of shell layers display great removal rates for the two model dyes used in this study according to the pseudo-second-order model. This present work suggests that diamond-based composites by LbL method can be promising candidates for dye adsorption and removal, which has great potentials to be applied to the field of wastewater treatment.

## Results and Discussion

**Figure 1:**
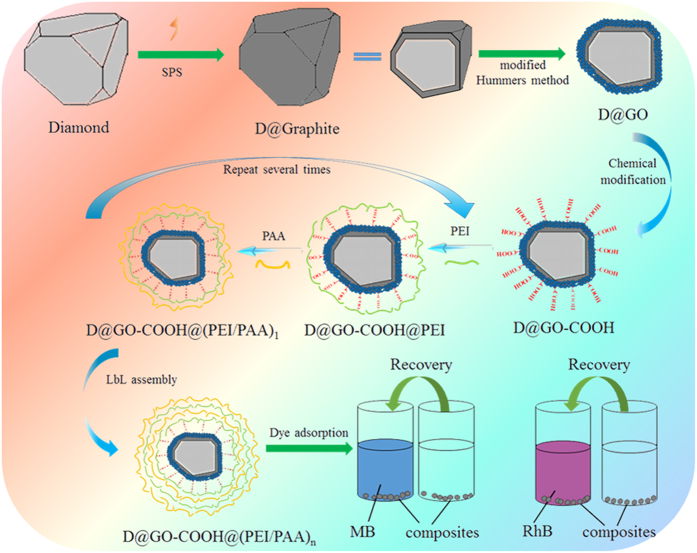
Schematic illustration of the fabrication of D@GO-COOH and LbL-assembled composites by chemical modification and LbL self-assembly.

Figure 1 depicts the 3-step experimental procedure, including the preparation of building block D@GO-COOH, the LbL assembly of the D@GO-COOH with PEI and PAA molecules, and the dye adsorption capacities of the obtained composites D@GO-COOH@(PEI/PAA)_n_. For the first step, to obtain core/shell structural D@G, diamond annealing was carried out in 10^−3^ Pa vacuum at the temperature of 1500 °C for 15 min to obtain graphite layer on diamond core. The D@GO was subsequently synthesized via the oxidation of graphite layer of D@G by utilizing H_2_SO_4_ and HNO_3_. After that, by reacting with chloroacetic acid, carboxyl groups were modified onto GO surface to obtain D@GO-COOH building block. The prepared D@GO-COOH was then used as a core part to construct hierarchical 3D porous structure with the LBL process via water soluble PEI and PAA molecules. The composites D@GO-COOH@(PEI/PAA)n were then obtained with the electronic force and hydrogen bond from -NH_3_^+^ and -COO^−^ groups. Two model dyes (i.e., MB and RhB) were then used to evaluate the adsorption capacity of the prepared composites.

**Figure 2:**
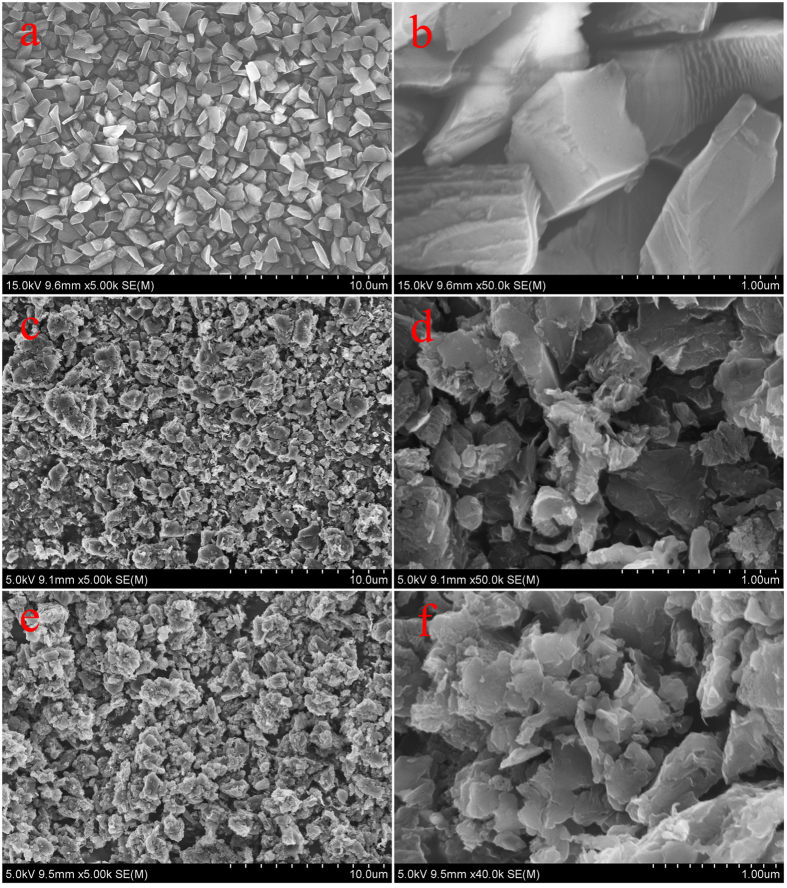
SEM images of as-prepared material powders: diamond (**a**,**b**), D@Graphite (**c**,**d**), and D@GO (**e**,**f**).

**Figure 3:**
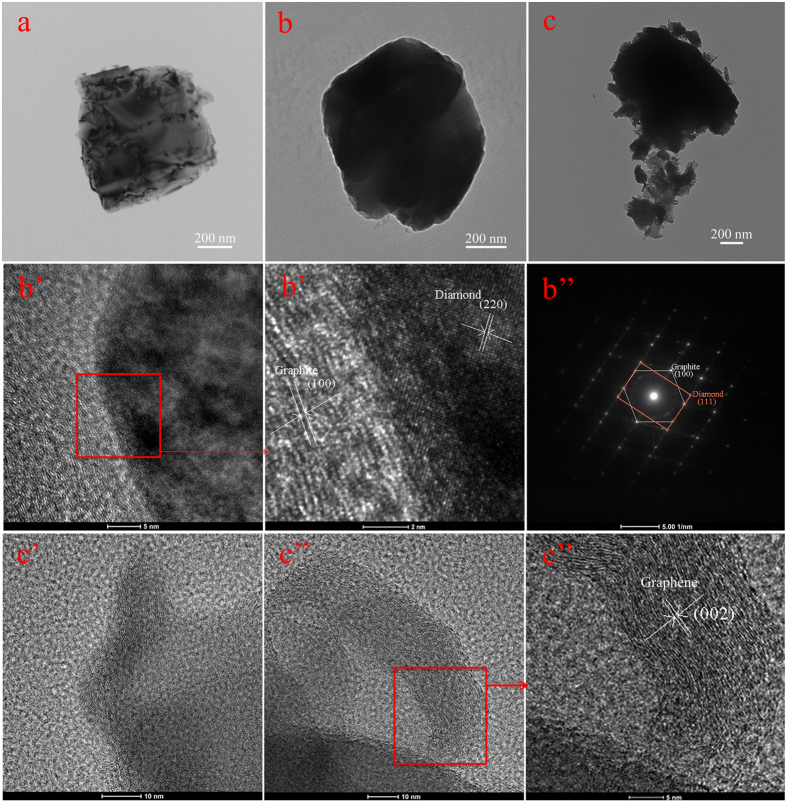
TEM images of as-prepared material powders: diamond (**a**), D@Graphite (**b**), and D@GO (**c**); HRTEM images of D@Graphite (b’) and D@GO (c’ and c”). Picture b” show the SAED patterns from picture b’.

**Figure 4:**
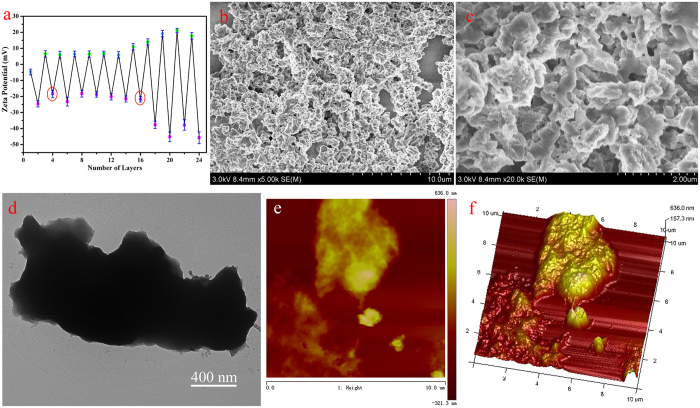
(**a**) Zeta potential data of LbL process; morphological characterization of the as-prepared D@GO-COOH@(PEI/PAA)_8_ composites: (**b** and **c**), SEM images; (**d**) TEM image; (**e** and **f**) AFM images in height and 3D modes.

SEM images (seen in Fig. 2) show the morphology of initial diamond, intermediate D@Graphite, and D@GO-COOH powders. In order to demonstrate the interfacial layer transition, TEM and HRTEM images were analyzed to characterize the morphology of D@Graphite and D@GO, as shown in Fig. 3. The TEM image of D@Graphite, displayed in Fig. 3b’, clearly demonstrates the interfacial coexistence of diamond crystal face (220) and graphite face (100), as well as the SAED patterns (seen in Fig. 3b”). The D@GO images (Fig. 3c’) present the crystal face of graphene (002), indicating successful modification of GO layer on the hierarchical diamond composite structures^15^. The elemental analysis data (Table 1) of as-prepared diamond-based composites show the obvious increment of oxygen and hydrogen composition, as well as the decrement of the carbon compositions of D@GO and D@GO-COOH, in comparison with intermediate D@Graphite material. Thus, we inspected the self-assembly process and characterized the prepared LbL-assembled core-shell composites through morphological and spectral methods. For the purpose of monitoring the LbL process, zeta potential (ζ) image in Fig. 4a presents the changes of surface charge with the alternative modification of the PEI and PAA on the surface of D@GO-COOH, which provides carboxyl groups for initial electrostatic interaction in LBL assembly. It can be easily observed that the values of zeta potential show stable state after one PEI/PAA bilayer cycle and vary at range of 5.8~6.7 mV for PEI modification and −18.8~−21.6 mV for PAA modification. With the modification layer increased onto 16 (i. e. 8 bilayer of LBL), big disturbance appeared due to poor dispersion in water solution mainly originated from the crosslinking shell composed of PEI/PAA between building blocks. Thus the obtained composite D@GO-COOH@(PEI/PAA)_8_ with lay number of 16 was chosen for the next nanostructural characterization. SEM characterization in Fig. 4b,c display that the structures of the composite D@GO-COOH/(PEI@PAA)_8_ show a granular arrangement with multilayered shell nanocomposites than the building block D@GO-COOH. TEM image in Fig. 4d confirm the assembled shell structure with jagged edge. In comparison with the AFM images D@GO-COOH (Figure S1), the images in height and 3D modes of composite D@GO-COOH/(PEI@PAA)_8_ in Fig. 4e,f show the fibrillar or web-like morphology of multilayered shell aggregates on the particle surface.

**Figure 5:**
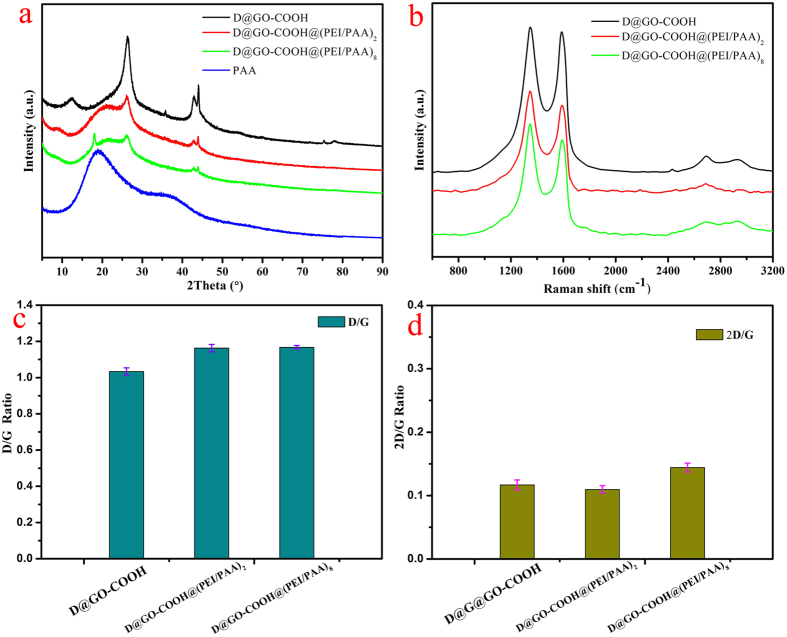
XRD curves (**a**) and Raman spectroscopy (**b**) of the prepared D@GO-COOH and LBL-assembled composites. Pictures **c** and **d** show the D/G ratios and 2D/G ratios in Raman data.

In addition, XRD was used to characterize the obtained organized nanostructures in the composite materials. Figure S2 shows the XRD curves of the synthesized intermediates and building blocks from initiative diamond. The peak with 2θ value of 43.92° can be corresponding to the (111) cubic diamond plane. The 2θ = 25.92° peak owns to the graphite’s (100) plane. The X-ray pattern of D@GO displays the presence of a strong peak at 11.67° corresponding to the (001) reflection peak with a layer distance of 0.76 nm. This clearly indicates the formation of GO layer on the graphite surface of D@Graphite. After modification with carboxyl groups, the relative intensity of this peak decrease obviously with a little shift to 12.47° in D@GO-COOH. Next, after LBL-assembled process, as shown in Fig. 5a, there appears a wide convex protrusion in the range of 15°–23° for the obtained D@GO-COOH@(PEI/PAA)_2_ composite. With the subsequent increment of LBL bilayer number, an acute peak at 18.08° appear in curve of D@GO-COOH@(PEI/PAA)_8_ composite, which can be assigned to the assembled PEI/PAA multilayer shell structures due to the formation of strong intermolecular hydrogen bonds and electronic forces in the presence of free amino and carboxyl groups in polymeric molecules. The above obtained XRD results clearly indicated that the hierarchical core-shell self-assembled composites have been successfully prepared.

It is well known that Raman spectroscopy provides a useful tool to characterize the carbon-based materials^34^. As shown in Figure S3a and Fig. 5b, three characteristic bands of graphene sheets in Raman spectra appear for the obtained composites, including the G band (1601 cm^−1^) originated from the first-order scattering of the E_2_g phonons of the sp^2^-hybridized carbon atoms, the D band (1351 cm^−1^) caused by a breathing mode of K-point phonons of A_1g_ symmetry of the defects involved in the sp^3^-hybridized carbon bonds such as hydroxyl and/or epoxide bonds, and the 2D band (2692 cm^−1^) which is much sensitive to stacking of graphene sheets^35^,^36^. In addition, the D/G ratio of peak intensity can serve as a characteristic factor of the sp^2^ area size of graphene sheets containing sp^3^ and sp^2^ bonds^37^,^38^. In comparison with the D/G ratio of about 0.22 for D@Graphite, obvious increment for D@GO and D@GO-COOH with values of 1.0 and 1.03 are easily observed in Figure S3b, indicating fewer defect layers in D@Graphite. And after LBL-assembled process, D@GO-COOH@(PEI/PAA)_2_ and D@GO-COOH@(PEI/PAA)_8_ composites demonstrate the increased D/G ratio values of 1.16 and 1.17 (Fig. 5c). Moreover, the 2D/G ratios for graphene sheets with different layers (1, 2, 3, >4) are normally >1.6, 0.8, 0.30, and 0.07, respectively^39^,^40^. For example, Akhavan and co-workers have reported single and bilayer GO sheets with 2D/G ratios in the range of 1.53–1.68 and 0.82–0.89, respectively^41^. For the present study, the 2D/G ratios in all composite materials were calculated to show values ranging from 0.11 to 0.14 (Figure S3c and Fig. 5d), indicating the formation of multilayer GO layer structures in present obtained composite materials.

**Figure 6:**
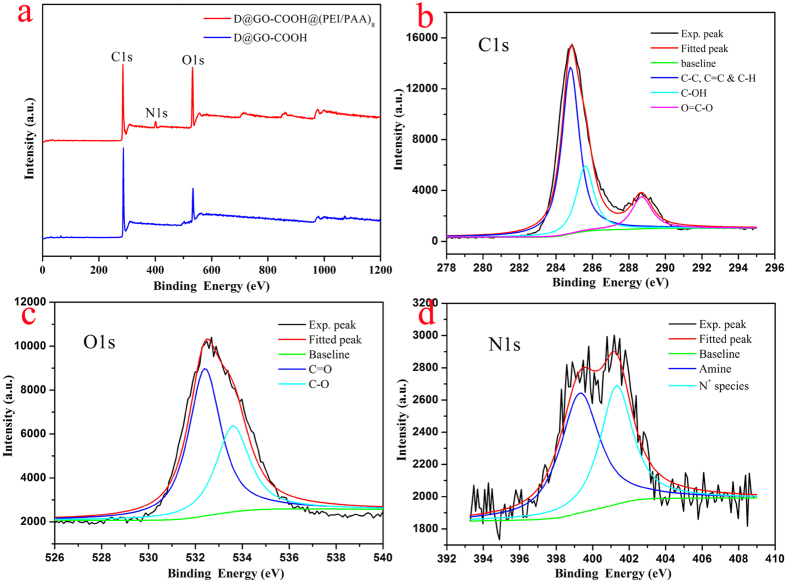
Survey XPS spectra of lyophilized composites D@GO-COOH and D@GO-COOH@(PEI/PAA)_8_ (**a**); Deconvolution of XPS peaks of the core-shell composite D@GO-COOH@(PEI/PAA)_8_: (**b**), C(1s); (**c**), O(1s); (**d**), N(1s).

Since the obtained core-shell diamond-based composites were designed and prepared for the adsorption purpose, it is important to perform interfacial analysis and composition analysis using XPS technique. First of all, the survey data of XPS spectra from D@GO-COOH and D@GO-COOH@(PEI/PAA)_8_ composites (Fig. 6a) demonstrate the characteristic peaks, such as C(1s), O(1s), and N(1s). And the relative elemental analysis data (Table 1) demonstrate the composition appearance of nitrogen element with the value of 5.74 wt.%, indicating the successful modification of PEI molecules in the shell layers via LbL assembly. In addition, the deconvolution of C(1s), O(1s), and N(1s) peaks for the D@GO-COOH@(PEI/PAA)_8_ composite material were analyzed and demonstrated. For the peak deconvolution of C(1s) core levels in Fig. 6b, the peak centered at 284.8 eV could be assigned to the C-C, C=C and C-H bonds. In addition, the other deconvoluted peaks at positions of 285.6 and 288.7 eV were attributed to C-OH and O=C-O oxygen-containing bonds, respectively^42^,^43^. Moreover, the O(1s) peak (Fig. 6c) could be deconvoluted into two main Gaussian component peaks after subtraction treatment of Shirley background. The first component peak centered at 533.6 eV could be assigned to the oxygen of C-O in the composite. The second deconvoluted O(1s) peak centered at 532.4 eV was assigned to the oxygen of C=O in composites. This means that the surface of core-shell composite was still functional and porous, which show an advantage for next adsorption application. Furthermore, the N(1s) peak (Fig. 6d) indicated the appearance of amine group at about 399.3 eV and N^+^ segments at about 401.3 eV, demonstrating the self-assembly of PEI molecules in composite via both covalent bonding and weak interaction force to the PAA and GO sheets.

**Figure 7:**
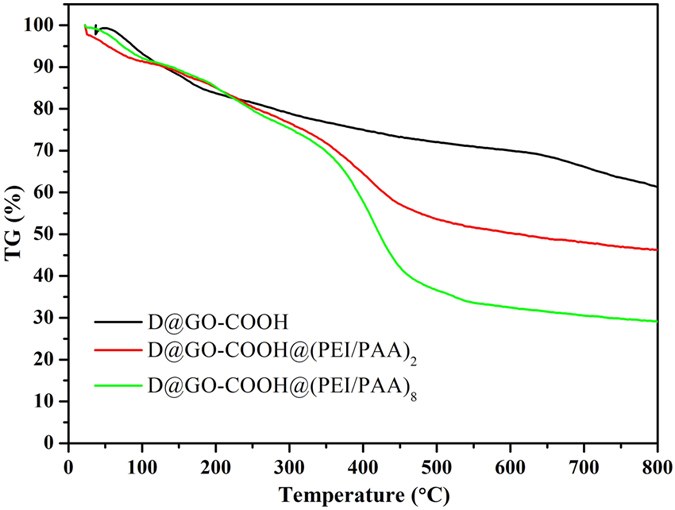
TG curves of D@GO-COOH and LbL-assembled composites.

The thermograms (TG) of the lyophilized as-synthesized intermediates and LbL-assembled composites are demonstrated in Fig. 7 and Figure S6. It can be clearly observed that the quality of D@Graphite keep invariable from room temperature to 800 °C with the quality reservation of 99.2%. As for the D@GO, with temperature increment, the quality of samples declines sharply in the range from 170 to 230 °C and subsequently decreases slowly until the quality reservation of 59.5%. After functionalization with carboxyl groups, D@GO-COOH as building block shows a little higher thermal stability than D@GO. Next, with the LbL assembled process, the obtained composites D@GO-COOH@(PEI/PAA)_2_ and D@GO-COOH@(PEI/PAA)_8_ display sharp quality-decreased range of 350–490 °C, which can be owing to the pyrolysis of the unstable oxygen containing functional groups and alkyl chains in polymeric molecules^44^,^45^,^46^,^47^. The final values of quality reservation at 800 °C are 46.3% and 29.1% for the 2-bilayer and 8-bilayer self-assembled composite materials. The above obvious changes of thermal stability demonstrate the successful modification of diamond and the formation of 3D hierarchical core-shell composite structures via LBL-assembled process.

**Figure 8:**
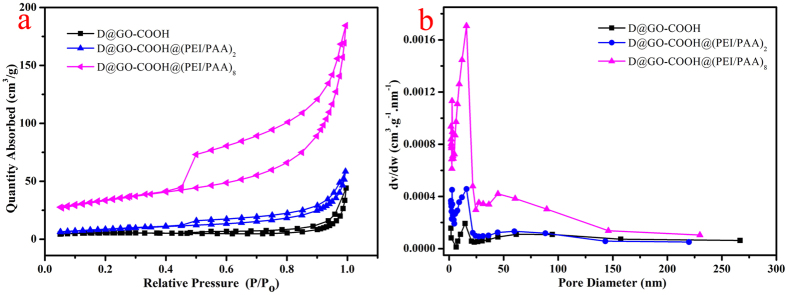
N_2_ adsorption–desorption isotherms (**a**) and pore size distribution (**b**) of D@GO-COOH and LBL-assembled composites.

The microstructural characteristics of as-prepared composites were further investigated with the N_2_ adsorption–desorption isotherms (Fig. 8 and Figure S7). The characteristics of type IV isotherms for composite D@GO-COOH@(PEI/PAA)_8_ were demonstrated, and a significant hysteresis loop at high relative pressure could be seen (P/P_0_ of 0.45–0.95), thereby indicating that the sample was mesoporous material. The hysteresis loops of D@GO-COOH and D@GO-COOH@(PEI/PAA)_2_ could be presented at higher relative pressure (P/P_0_ of 0.85–0.95). The pore size distribution curves were calculated by BJH methods, and the properties of the samples were summarized in Table 2 and Table S1. The pore size for D@GO-COOH and LbL-assembled composites were 15–20 nm (Fig. 8b). The 3D hierarchical porous structure of D@GO-COOH@(PEI/PAA)_8_ could adsorb more nitrogen and had a relatively high BET specific surface area of 114.5571 m^2^g^−1^, which was much larger than that of D@GO-COOH (20.3475 m^2^g^−1^). Furthermore, the pore volume and average pore diameter were also larger than that of D@GO-COOH. Higher specific surface areas can increase the number of adsorbent activity points as well as the chance of contact between pollutant molecules and the active point. Larger pore diameters and pore volumes can offer abundant channels for the dye solution and reduce the mass transfer resistance of the reaction process, thereby making it easier for the pollutant molecules to reach the active points, which improves the absorption performance.

In order to evaluate the successful functionalization on diamond and characterize the prepared LBL-assembled composites, FT-IR spectra were also measured. The spectra of D@GO and D@GO-COOH (Figure S8) show typical bands due to skeletal vibrations of graphene domains at 1621 cm^−1^ and characteristic OH and C=O stretching at 3438 and 1723 cm^−1^ as well as epoxy C–O (1226 cm^−1^) and alkoxy C–O (1045 cm^−1^) groups^48^,^49^, which indicate the effective formation of GO layer and functionalization of carboxyl groups. In addition, obvious changes were observed for the IR spectra of prepared LBL-assembled composite D@GO-COOH@(PEI/PAA)_8_ with polymeric shell structure. The spectrum show obvious intensity for bands at 3448 cm^−1^ (NH and COOH stretching), 2921 and 2850 cm^−1^ (C-H modes of methylene moieties), 1719 cm^−1^ (C=O stretching), 1627 cm^−1^ (amide stretching), and 1464 cm^−1^ (scissoring of methylene chains), indicating the polymeric shell formation with PEI and PAA molecules in the assembled composite materials.

**Figure 9:**
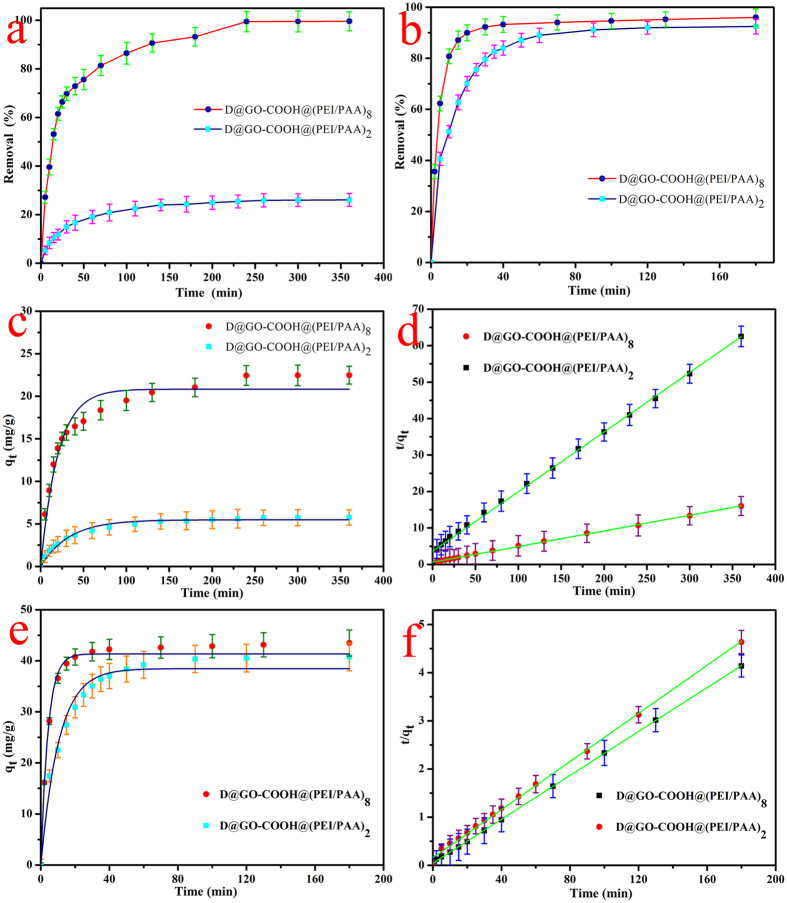
The percent dye removal rate versus time plots and adsorption kinetics curves of as-prepared D@GO-COOH@(PEI/PAA)_2_ and D@GO-COOH@(PEI/PAA)_8_ composites on RhB (**a**,**c**,**d**), and MB (**b**,**e**,**f**) at 298 K.

Next, the dye adsorption capacity was evaluated by placing the as-prepared self-assembled diamond-based composites in MB and RhB aqueous solutions. Different absorbance wavelengths (664 nm for MB, and 554 nm for RhB) were utilized to investigate the residual dye concentrations at various time intervals. At the same time, the dye removal rates were calculated according to the equation: K = (A_0_ − A_T_)/A_0_ × 100%, where K is defined as the dye removal rate, A_0_ is defined as the absorbance of the dye solution, and A_T_ is defined as the absorbance of the supernatant liquid collected at different intervals. Figure 9a,b show dye removal rate versus time plots for the two used dyes RhB and MB. The dye removal rates reach stable values for RhB and MB within approximately 240 min and 80 min, suggesting that the as-prepared composites act as efficient dye adsorbents. Adsorption kinetic experiments of the as-prepared nanocomposites were performed with the results for RhB (Fig. 9c,d) and MB (Fig. 9e,f). In order to compare the adsorption performance of self-assembled composites, the adsorption properties of self-assembling building block D@GO-COOH were also characterized (Figure S9). In addition, classical kinetic models were used to demonstrate the above degradation mechanism as follows:

The pseudo-first-order model can be represented by equation (1):


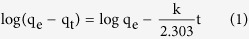


**Figure 10:**
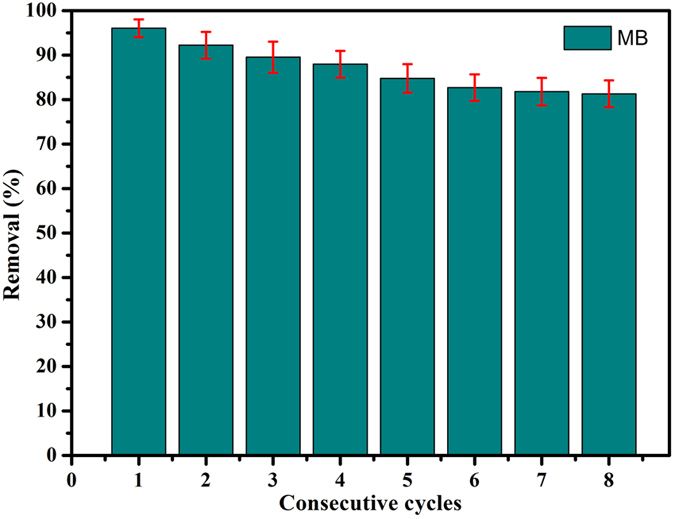
Relative adsorption capacity and regeneration studies of as-prepared D@GO-COOH@(PEI/PAA)_8_ composite towards MB at room temperature for different consecutive cycles.

The pseudo-second-order model can be represented by equation (2):


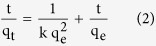


where q_e_ and q_t_ demonstrate the amount of dye adsorbed (mg/g) at equilibrium and time t, respectively, and the k_1_ and k_2_ values are the kinetic rate constants^50^,^51^. The kinetic results (Table 3 and Table S2) can be well characterized by pseudo-second-order model with good correlation coefficients (R^2^ > 0.995). On the other hand, good recovery and stability are expected and preferable for the large-scale applications of composite absorbents. In our recent study, the facile preparation and the dye removal capacity of GO/Fe_3_O_4_ nanocomposites and core–shell MnO_2_ nanocomposites have been characterized and investigated in details, in which the obtained composite materials can be easily reused and recycled several times, suggesting the long term stability and application prospect in water purification^52^,^53^. Here, the as-prepared D@GO-COOH and LBL-assembled composite materials were used to investigate the potential application for dye removal. Unlike other reported nanoparticle absorbents, the obtained composite absorbents can be easily separated from wastewater solution. After maximum adsorption process in dye solutions, the as-prepared D@GO-COOH@(PEI/PAA)_8_ composite were treated by thoroughly cleaning procedures to remove possible dye residues and to regenerate the materials. The adsorption experiments were repeated for eight consecutive cycles using the same composite and fresh MB solution (Fig. 10). The results indicate that the removal rate towards MB maintains at about 81.3% after eight consecutive cycles compared to 96.1% m in the first adsorption process for D@GO-COOH@(PEI/PAA)_8_ composite, demonstrating an excellent stability and reutilization of the obtained composite materials in this study. In addition, further recycling steps indicated slight decrement of adsorptive efficiency, which could be attributed to the byproduct deposition on composite surfaces or slight loss of composites by many washing steps. The above reused data suggested that the prepared composite materials can be potentially applied for wastewater treatment.

In summary, we have successfully constructed diamond-based core-shell composites D@GO-COOH@(PEI/PAA)_n_ from chemically-modified diamond via LbL self-assembly have been designed and developed. Because of the characteristic high surface area and chemical groups in the obtained composites with the hierarchical core-cell structures, the obtained GO-COOH@(PEI/PAA)_n_ composites showed efficient adsorption capacity towards the two model dyes used in this study. In addition, the as-prepared composite materials can be easily separated from wastewater dye solution and demonstrate excellent reusability. The present work is expected to open a new avenue for the design and preparation of diamond functionalization and self-assembled core-shell composites.

## Methods

Microdiamond powders were supplied by Element Six Ltd (Henan, China). Polyethylenimine (abbreviated as PEI, 99% hydrolyzed, average M.W. 600), poly acrylic acid (abbreviated as PAA, M.W. ~450,000), and chloroacetic acid were purchased from Aladdin Reagent (Shanghai, China) and Alfa Aesar Chemicals (Shanghai, China). The ammonia water (NH_3_ · H_2_O) and ethanol (C_2_H_5_OH) were provided from Sinopharm Chemical Reagent Co., Ltd (Beijing, China). Rhodamine B (abbreviated as RhB) and methylene blue (abbreviated as MB) were obtained from Tianjin KaiTong Chemical Reagent co., Ltd (Tianjin, China) and used without further purification. Sulfuric acid (H_2_SO_4_, 98%), potassium permanganate (KMnO_4_), potassium nitrate (KNO_3_), hydrogen peroxide (H_2_O_2_, 30%, w/w), and hydrochloric acid (HCl, 37%) were purchased from Beijing Chemicals, and used without further purification. All aqueous solutions were prepared with water purified in a double-stage Millipore Milli-Q Plus purification system.

The diamond powder (10.0 g) with diameter range of 1–2 micron was washed by ammonia water (NH_3_ · H_2_O, 25–28 wt.%) for three times under ultrasonic bath, and subsequently filtered the solution using ethanol (95 wt.%). Next, the dried diamond powder was obtained by heating in oven at 80 °C. With the temperature of 1500 °C, the diamond agglomerate was carried out by the vacuum spark plasma sintering (SPS) for 15 minutes. Finally, the synthesized diamond@graphite (abbreviated as D@Graphite) powder (9.2 g) was obtained by grinding the agglomerates.

Diamond@graphene oxide (abbreviated as D@GO) was synthesized from D@Graphite powder as raw material by a modified Hummers method^54^. In this method, H_2_SO_4_ (50 mL) was poured into a beaker and cooled to 0 °C. And D@Graphite powder (1 g) and KNO_3_ (1.5 g) were added to the cooled H_2_SO_4_ with strong mechanical stirring throughout the preparation process. KMnO_4_ (8 g) was added slowly into the mixed suspension after stirring for 30 min. Then the mixture was reacted at 35 °C for additional 6 h. After that, 200 mL of deionized water was added slowly into the mixture, and then the temperature was kept at 80 °C for 30 min. H_2_O_2_ (30%, 20 mL) was used to remove residual KMnO_4_ after diluting the mixture with 200 mL deionized water. Then the 5 wt% HCl aqueous solution was used to remove the redundant ions in the mixture. For purification, the mixture was washed with deionized water and centrifuged several times. Finally, the designed D@GO material was synthesized under freeze-dried at low temperature (−50 °C).

The carboxyl-functionalized D@GO (named as GO-COOH) was prepared according to the relative reported literature^55^. The D@GO powder (400 mg) was mixed with 400 mL cooled deionized water in a beaker at 0 °C under mechanical stirring for 30 min throughout the whole preparation process. Then ClCH_2_COOH (6 g) was added slowly into the mixed suspension during stirring. NaOH solution (6 mol/L, 32 mL) was added to remove residual chemical groups. The mixture was reacted at 70 °C for 3 h. After that, in order to improve the purification, the mixture was washed with deionized water and centrifuged several times. Finally, the carboxyl-terminated microdiamond D@GO-COOH was obtained under freeze-dried in low temperature (−50 °C).

The obtained D@GO-COOH (10 mg) was put into a plastic test tube, and 1 mL aqueous PEI solution (100 mg/mL) was added and kept with magnetic stirring for 1 h. Thus, D@GO-COOH@PEI was obtained by washing 7–8 times using deionized water and centrifugation. After that, 1 mL aqueous PAA solution (75 mg/mL) was poured into the D@GO-COOH@PEI with magnetic stirring for 1 h. After that, D@GO-COOH@(PEI/PAA)_1_ was obtained by similar washing 7–8 times using deionized water and centrifugation. The Layer-by-layer (LbL) assembly process for D@GO-COOH was achieved by the cyclic operation of the above procedures of PEI and PAA. So the D@GO-COOH@(PEI/PAA)_n_ (n = 1~12) composite materials with different bilayers was obtained due to the strong electronic force and hydrogen bonds between functional group of -NH_3_^+^ and -COO^−^. Finally, the composites were carried out under freeze-dried in low temperature (−50 °C).

Adsorption capacity experiments were designed and modified according to previous reports^56^,^57^. In adsorption experiments, about 10 mg of D@GO-COOH@(PEI/PAA)_n_ was added to two used dye solutions with volume of 50 mL (MB, 10 mg/L; RhB, 5 mg/L), respectively. The mixed dye solutions were stirred with continuous and uniform rate in dark condition at room temperature. Supernatant liquid was withdrawn at different time intervals for subsequent characterization using an UV-vis spectrometer (752-type, Sunny Hengping scientific instrument Co., Ltd, Shanghai, China). Different absorbance wavelengths (662 nm for MB, 554 nm for RhB) were displayed to character the concentration of residual dyes with the pre-established calibration curves, respectively. For the recycling experiments, the as-prepared D@GO-COOH@(PEI/PAA)_8_ composites (10 mg) were added into 50 mL MB solution (10 mg/L) under mild stirring. After 2 h of maximum adsorption, the composite materials were washed thoroughly with deionized water and ethanol for several times. The adsorption processes were repeated for eight consecutive cycles using the same composites and initial fresh MB solution.

All the present synthesized materials were obtained by lyophilizer at −50 °C temperature via a lyophilizer (FD-1C-50, Beijing Boyikang Experimental Instrument Co., Ltd., China) to completely remove water over 2–3 days. The morphologies were characterized by using a field-emission scanning electron microscope (SEM) (S-4800II, Hitachi, Japan) with 5–15 kV accelerating voltage as well as a transmission electron microscope (TEM) (HT7700, Hitachi High-Technologies Corporation, Japan). High resolution TEM pictures and corresponding selected-area electron diffraction (SAED) patterns were recorded on a Talos F200X TEM electron microscope (FEI Trading (Shanghai) Co., Ltd, China) operated at an accelerating voltage of 200 kV. Atomic force microscope (AFM) data were performed via a Nanoscope model Multimode 8 Scanning Probe Microscope (Veeco Instrument, USA) with silicon cantilever probes. Thermogravimetry-differential scanning calorimetry (TG-DSC) characterizations were carried out by using a NETZSCH STA 409 PC Luxxsi multaneous thermal analyzer (Netzsch Instruments Manufacturing Co, Ltd, Germany) in argon gas atmosphere. X-ray diffraction (XRD) was conducted on an X-ray diffractometer equipped with a Cu Kα X-ray radiation source and a Bragg diffraction setup (SMART LAB, Rigaku, Japan). FT-IR spectra were obtained by a Fourier infrared spectroscopy (Thermo Nicolet Corporation) via the KBr tablet method. Raman spectroscopy was measured via a Horiba Jobin Yvon Xplora PLUS confocal Raman microscope equipped with a motorized sample stage. The wavelength of the excitation laser was 532 nm and the laser power was maintained below 1 mW without noticeable sample heating. X-ray photoelectron spectroscopy (XPS) was measured on the Thermo Scientific ESCALab 250Xi using 200 W monochromated Al Kα radiation. The 500 μm X-ray spot was used for XPS analysis. The base pressure in the analysis chamber was about 3 × 10^−10^ mbar. Typically the hydrocarbon C1s line at 284.8 eV from adventitious carbon is used for energy referencing. Both survey scans and individual high-resolution scans for characteristic peaks were recorded. Elemental analysis was carried out with a Flash EA Carlo-Erba-1106 Thermo-Quest. The specific surface areas and pore diameter distribution were determined by the BET measurement (NOVA 4200-P, US). The LBL assembly of D@GO-COOH composites with PEI and PAA was analyzed by measuring zeta potential (ξ) for each step of self-assembly by using Nanozetasizer machine (ZEN 3690, Malvern Instruments, UK).

## Additional Information

**How to cite this article:** Zhao, X. *et al*. Fabrication of Hierarchical Layer-by-Layer Assembled Diamond-based Core-Shell Nanocomposites as Highly Efficient Dye Absorbents for Wastewater Treatment. *Sci. Rep.* **7**, 44076; doi: 10.1038/srep44076 (2017).

**Publisher's note:** Springer Nature remains neutral with regard to jurisdictional claims in published maps and institutional affiliations.

## Supplementary Material

Supplementary File

## Figures and Tables

**Table 1:** Elemental analysis data of as-prepared diamond-based materials.

Sample	N [wt.%]	C [wt.%]	H [wt.%]	O [wt.%]
D@Graphite	—	99.25	0.164	0.466
D@GO	—	64.57	1.74	32.77
D@GO-COOH	—	65.33	1.97	32.49
D@GO-COOH@(PEI/PAA)_8_	5.74	54.72	5.53	34.02

**Table 2:** Physical data of D@GO-COOH and LbL-assembled composites.

Samples	Specific surface area (m^2^g^−1^)	Average pore Diameter (nm)	Pore volume (cm^3^g^−1^)
D@GO-COOH	20.3475	16.40024	0.068284
D@GO-COOH@(PEI/PAA)_2_	30.7095	11.79437	0.090550
D@GO-COOH@(PEI/PAA)_8_	114.5571	65.24655	0.285320

**Table 3:** Kinetic parameters of the obtained LbL-assembled composites for RhB and MB removal at 298 K (experimental data from Fig. 9).

RhB	Pseudo-first-order model			Pseudo-second-order model		
	q_e_ (mg/g)	R^2^	K_1_ (min^−1^)	q_e_ (mg/g)	R^2^	K_2_ (g/mg · min)
D@GO-COOH@(PEI/PAA)_2_	5.4833	0.9771	3.02 × 10^−2^	6.1120	0.9968	7.38 × 10^−3^
D@GO-COOH@(PEI/PAA)_8_	20.8350	0.9577	4.98 × 10^−2^	23.3263	0.9980	2.96 × 10^−3^
**MB**						
D@GO-COOH@(PEI/PAA)_2_	38.4633	0.9844	9.54 × 10^−2^	40.0320	0.9952	3.99 × 10^−3^
D@GO-COOH@(PEI/PAA)_8_	41.3414	0.9981	2.41 × 10^−1^	43.8596	0.9997	1.20 × 10^−2^
