# Supplementary material for: Fabrication of Hierarchical Layer-by-Layer Assembled Diamond-based Core-Shell Nanocomposites as Highly Efficient Dye Absorbents for Wastewater Treatment
Source: Sci Rep. 2017 Mar 8;7:44076. doi: 10.1038/srep44076 (PMC5341016; doi:10.1038/srep44076)
Supplement: Supplementary File [file srep44076-s1.doc]

Supporting Information

**Fabrication of Hierarchical Layer-by-Layer Assembled Diamond-based Core-Shell Nanocomposites as Highly Efficient Dye Absorbents for Wastewater Treatment**

Xinna Zhao^1,2^, Kai Ma^2^, Tifeng Jiao^1,2^, Ruirui Xing^2,3^, Xilong Ma^1^, Jie Hu^2^, Hao Huang^1^, Lexin Zhang^2^ & Xuehai Yan^3^

^1^State Key Laboratory of Metastable Materials Science and Technology, Yanshan University, Qinhuangdao 066004, China

^2^Hebei Key Laboratory of Applied Chemistry, School of Environmental and Chemical Engineering, Yanshan University, Qinhuangdao 066004, China.

^3^State Key Laboratory of Biochemical Engineering, Institute of Process Engineering, Chinese Academy of Sciences, Beijing 100190, China

Correspondence and requests for materials should be addressed to T.J. (email: tfjiao@ysu.edu.cn) or J.H. (email: hujie@ysu.edu.cn)

**Table S1.** Physical data of the obtained D@Graphite and D@GO composites

| Sample | Specific surface area (m^2^ g^-1^) | Average pore  Diameter (nm) | Pore volume  (cm^3^ g^-1^) |
| --- | --- | --- | --- |
| D@Graphite | 16.6545 | 11.99719 | 0.069532 |
| D@GO | 25.9754 | 14.10831 | 0.077908 |

**Table S2.** Kinetic parameters of the obtained D@GO-COOH for RhB and MB removal at 298 K (experimental data from **Figure S9**).

| D@GO-COOH | Pseudo-first-order model | | | Pseudo-second-order model | | |
| --- | --- | --- | --- | --- | --- | --- |
|  | q_e_  (mg/g) | R^2^ | K_1_  (min^-1^) | q_e_  (mg/g) | R^2^ | K_2_  (g/mg⋅min) |
| RhB | 37.666 | 0.9752 | 7.67 × 10^-2^ | 41.666 | 0.9997 | 3.33 × 10^-3^ |
| MB | 3.8375 | 0.9830 | 3.72 × 10^-2^ | 4.2323 | 0.9980 | 1.32 × 10^-2^ |


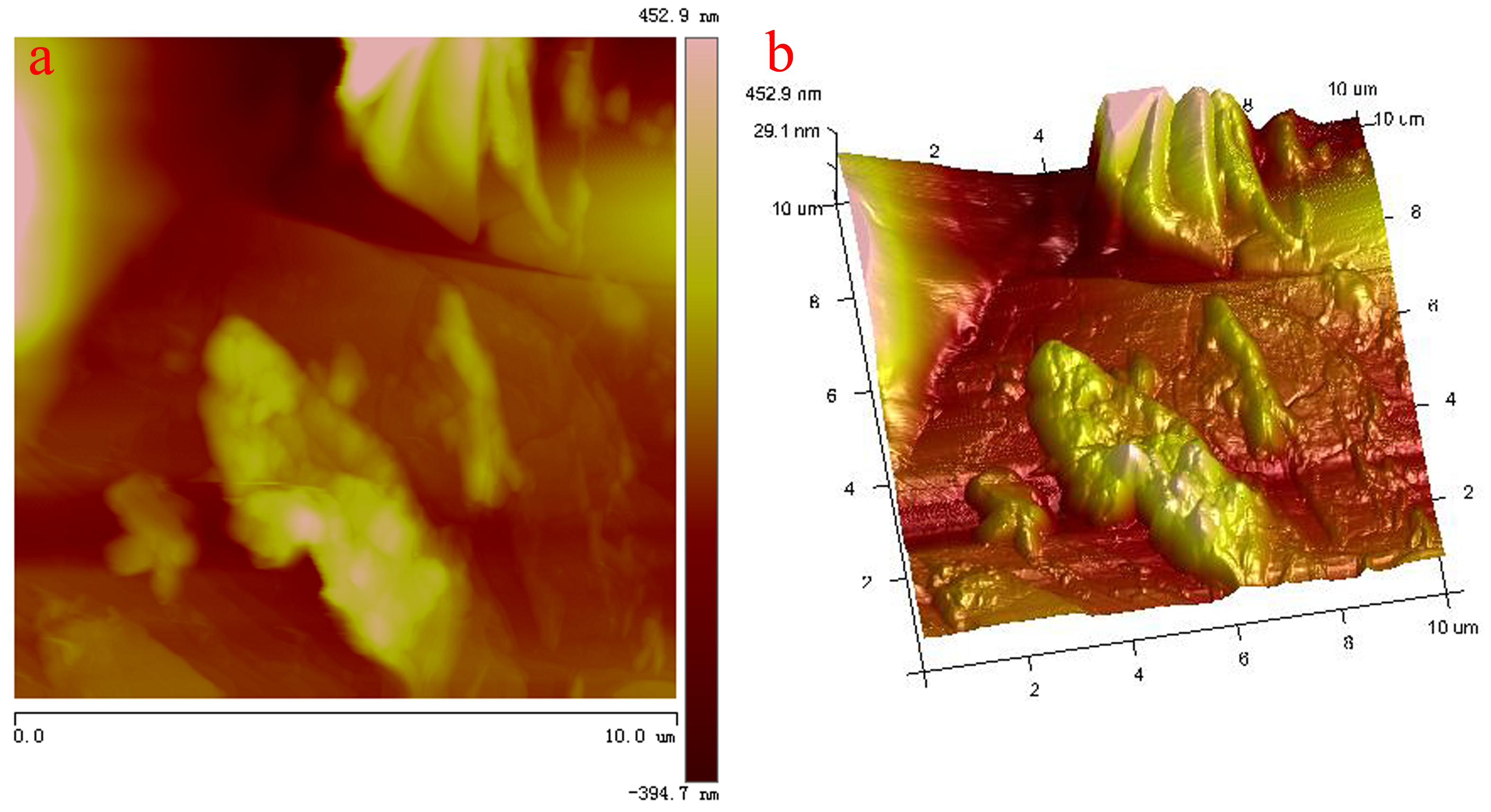


**Figure S1.** AFM images of the lyophilized D@GO-COOH composite material with height model (a) and 3D model (b).


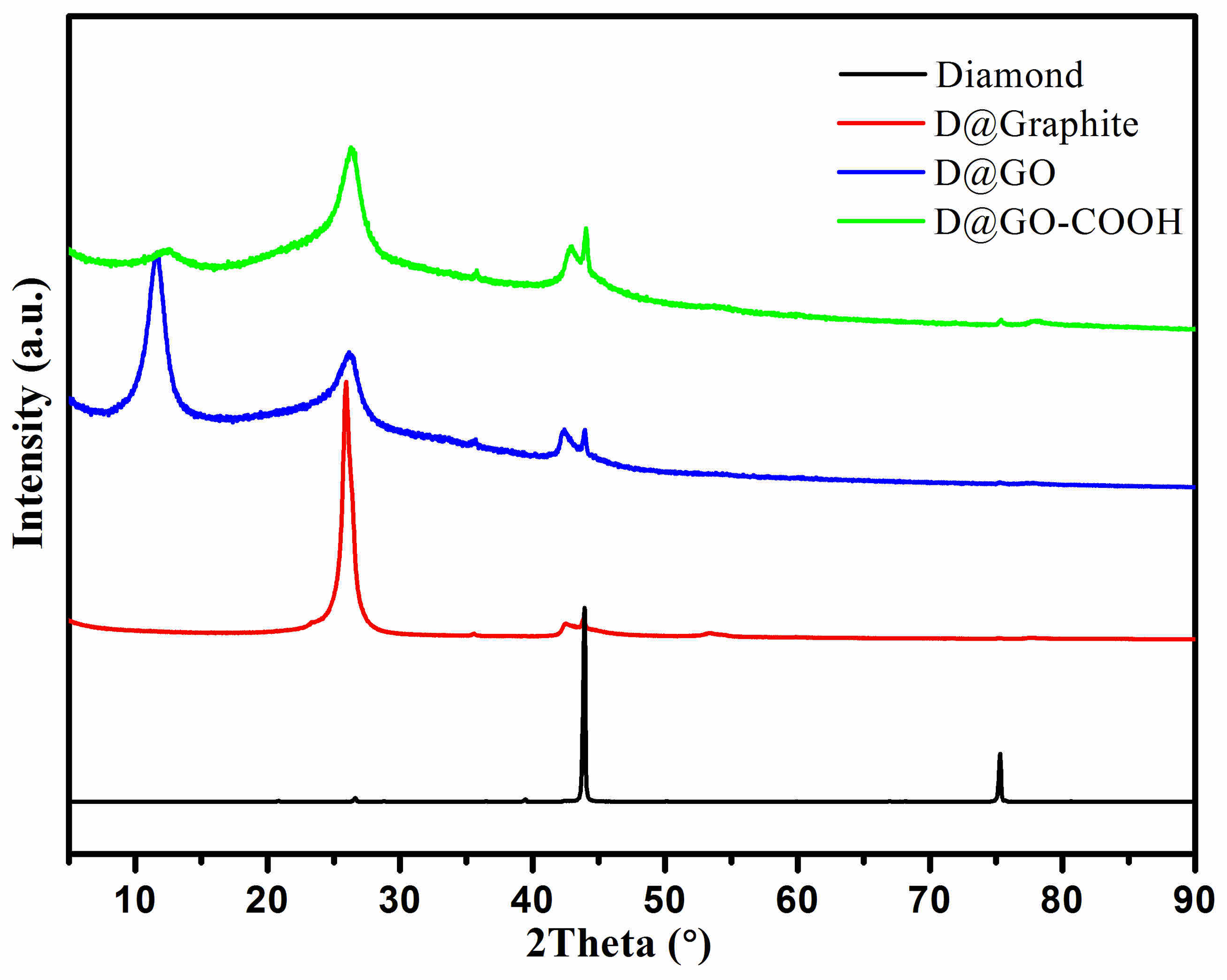


**Figure S2.** XRD curves of as-prepared materials: diamond, D@Graphite, D@GO, and D@GO-COOH.


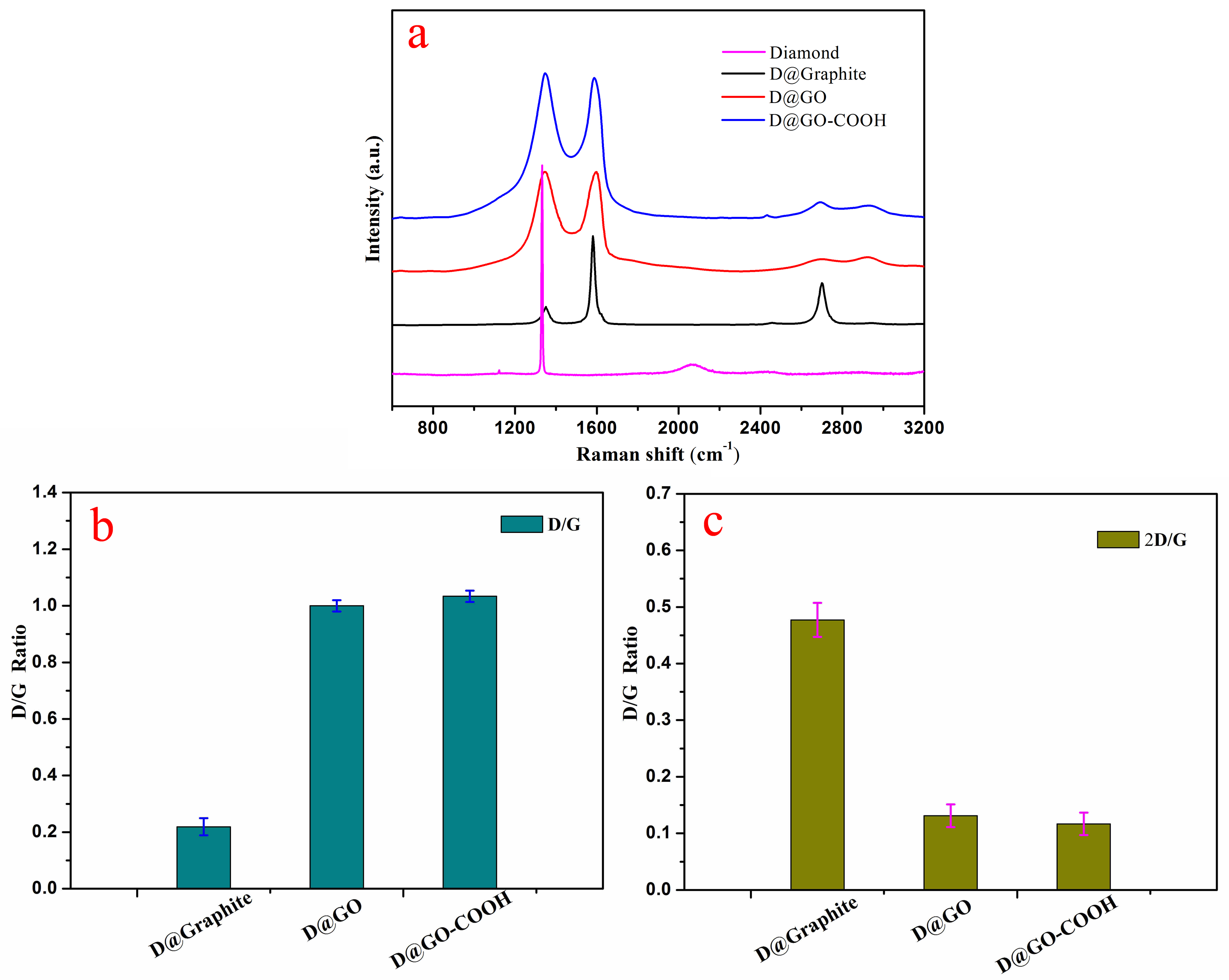


**Figure S3.** Raman spectroscopy (b), D@G ratios (c), and 2D@G ratios (d) of the as-prepared materials.


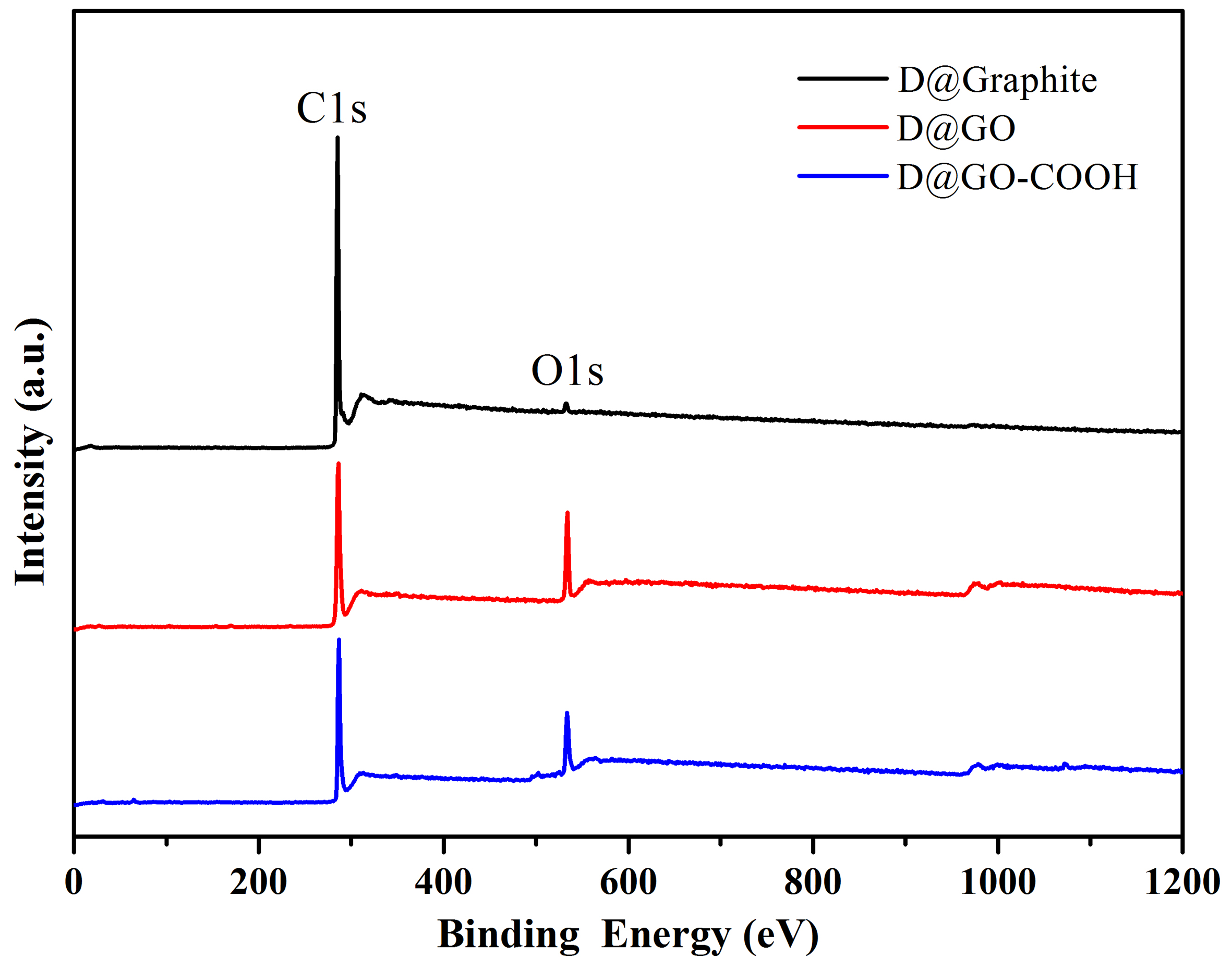


**Figure S4.** Survey XPS spectra of the as-prepared materials: D@GO-COOH, D@GO, and D@Graphite.


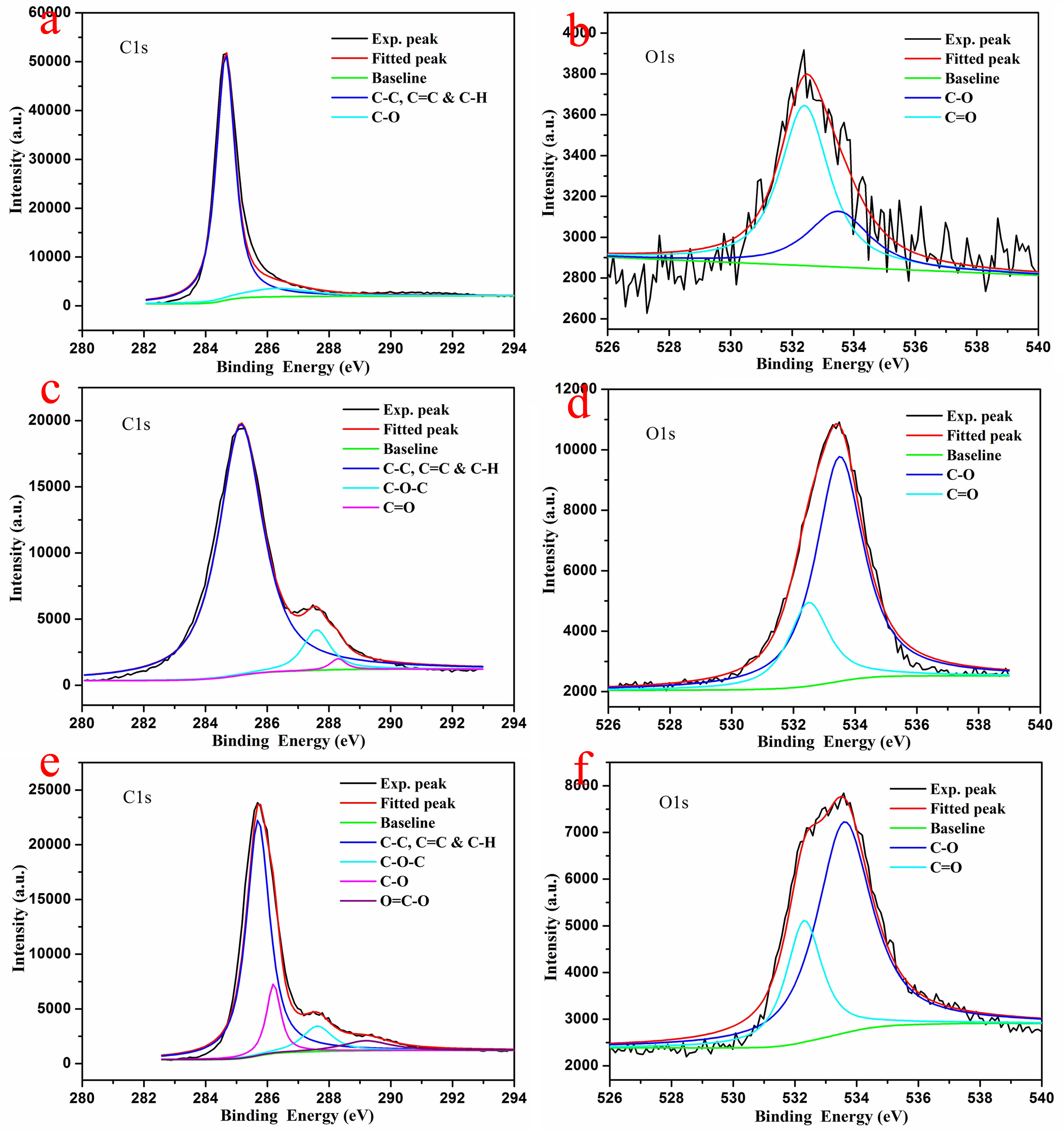


**Figure S5.** Deconvolution of XPS C(1s) peaks of D@Graphite (a), D@GO (c), and D@GO-COOH (e); O(1s) peaks of D@Graphite (b), D@GO (d), and D@GO-COOH (f).


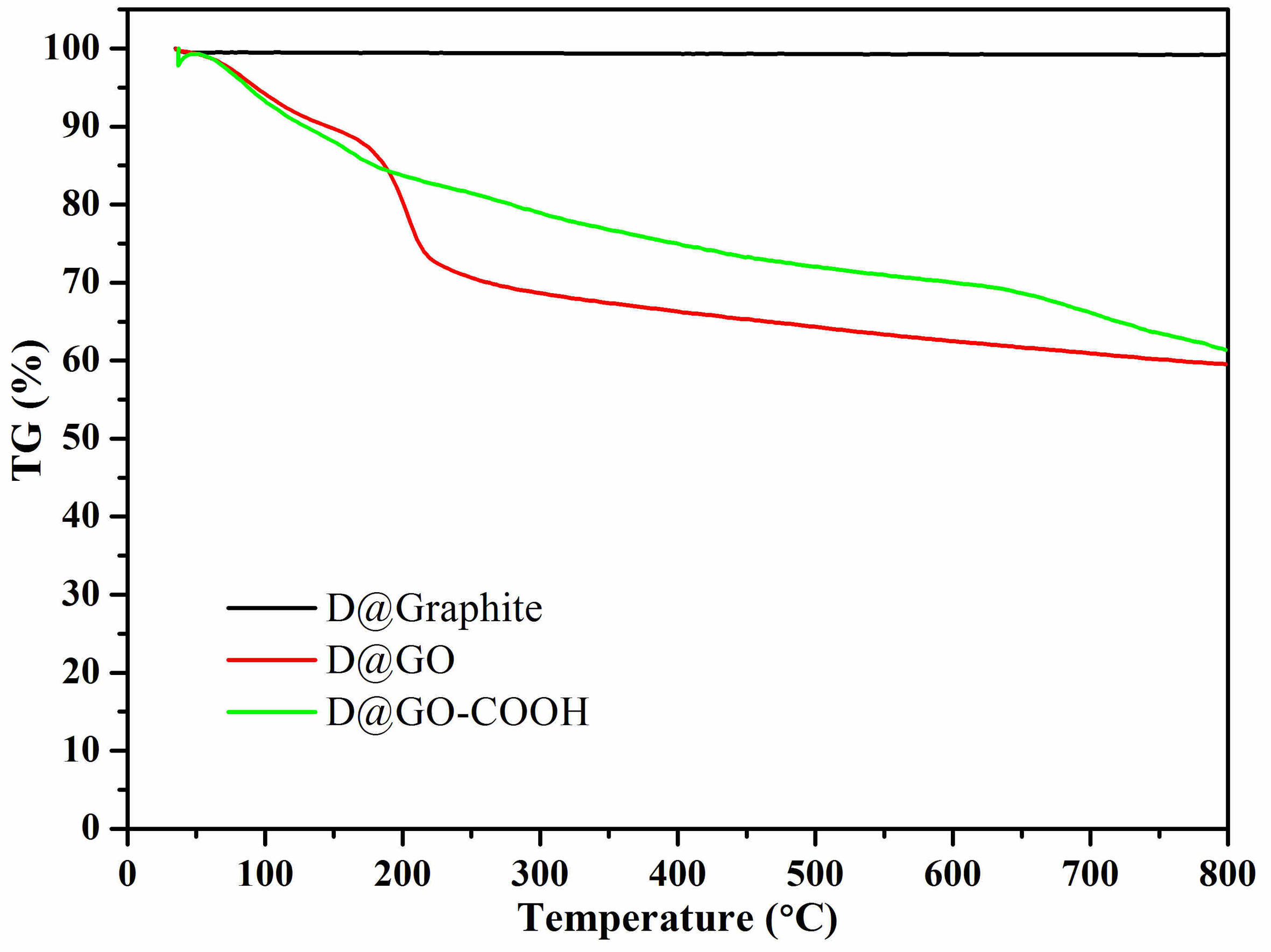


**Figure S6.** TG curves of as-obtained composite materials: D@GO-COOH, D@GO, and D@Graphite.


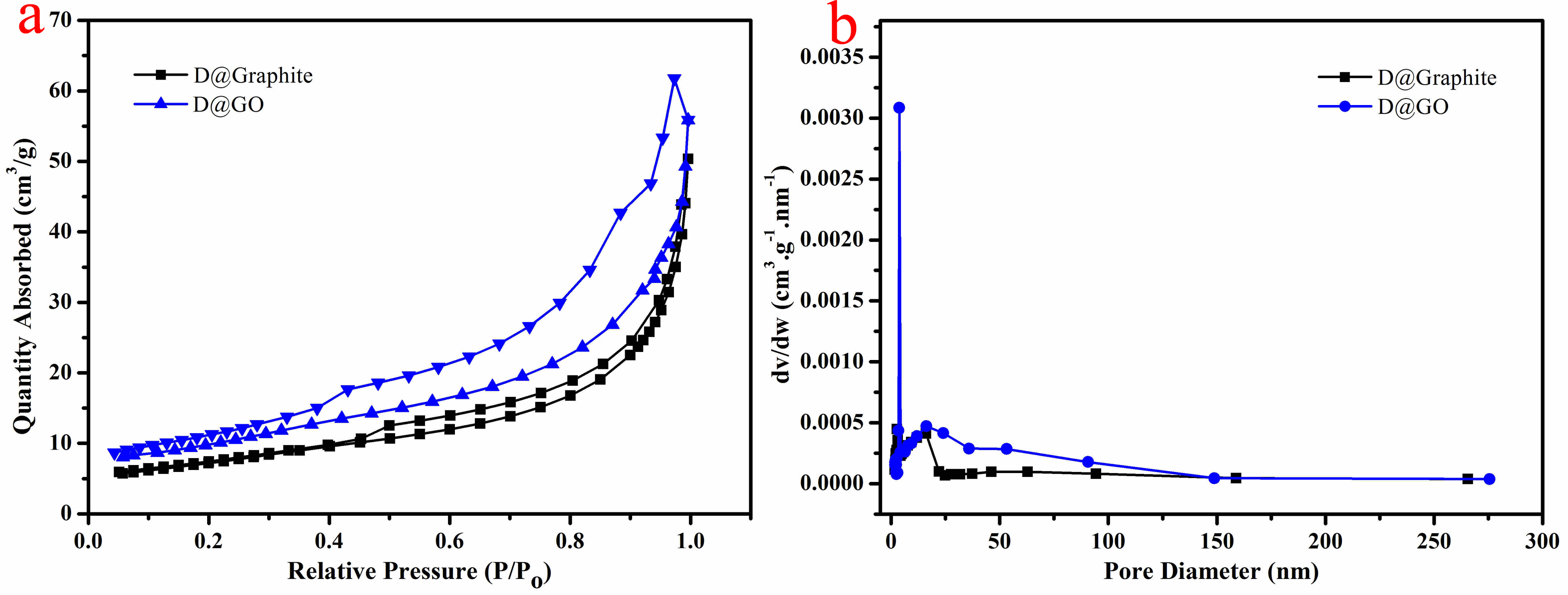


**Figure S7.** N_2_ adsorption–desorption isotherms (a) and pore size distribution (b) of D@Graphite and D@GO.


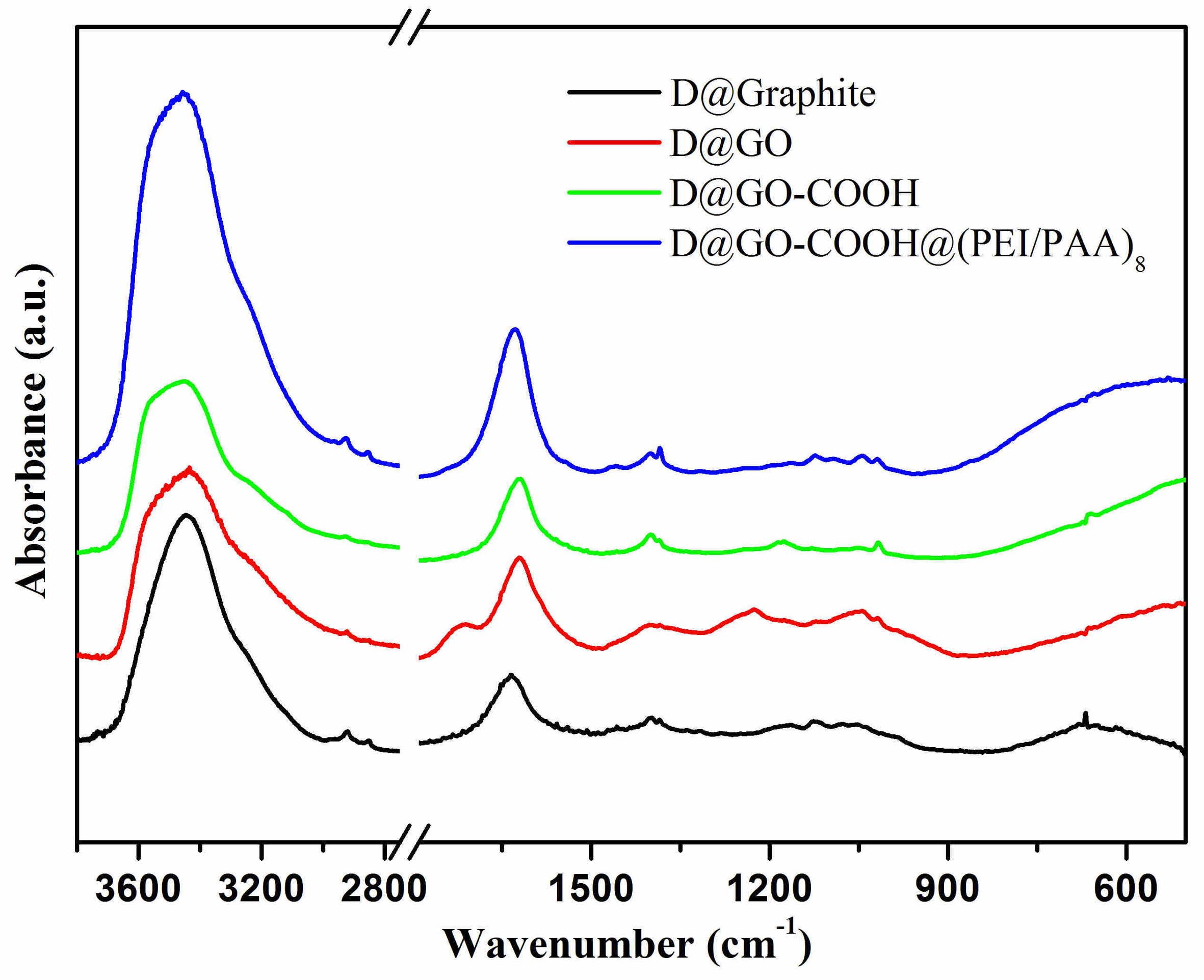


**Figure S8.** IR spectra of as-prepared materials: D@GO-COOH@(PEI/PAA)_8_, D@GO-COOH, D@GO, and D@Graphite, respectively.


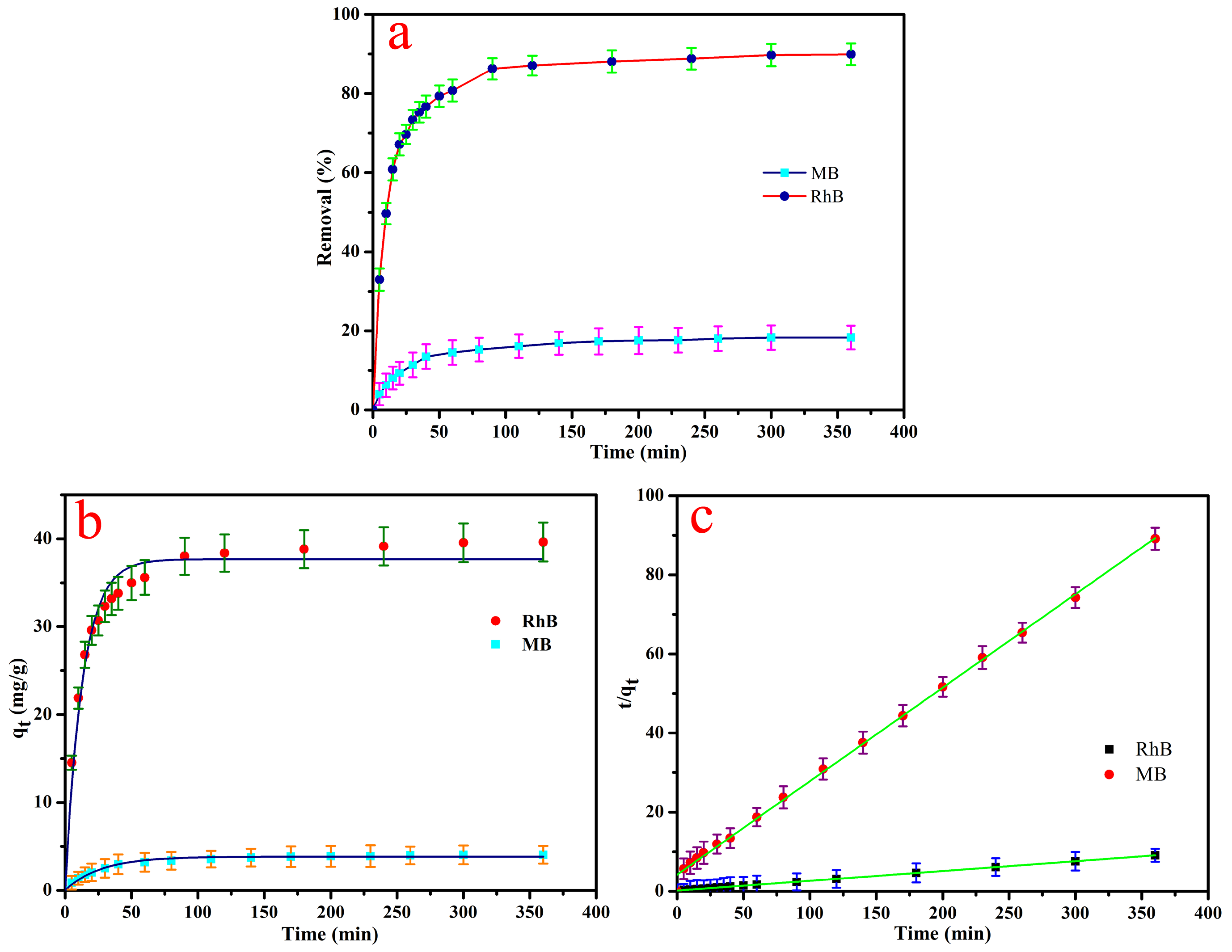


**Figure S9.** Adsorption kinetics curves of as-prepared D@CO-COOH on MB and RhB at 298 K: a, adsorption removal rate versus time plots; b, pseudo-first-order kinetics; c, pseudo-second-order kinetics.
